# The evolving landscape of ER-LD contact sites

**DOI:** 10.3389/fcell.2024.1483902

**Published:** 2024-10-03

**Authors:** Akhil Kumar, Surabhi Yadav, Vineet Choudhary

**Affiliations:** Department of Biotechnology, All India Institute of Medical Sciences (AIIMS), New Delhi, India

**Keywords:** lipid droplets, organelle biogenesis, neutral lipid storage, ER-LD contact site, seipin, lipid storage disorders, fatty acids, lipotoxicity

## Abstract

Lipid droplets (LDs) are evolutionarily conserved dynamic organelles that play an important role in cellular physiology. Growing evidence suggests that LD biogenesis occurs at discrete endoplasmic reticulum (ER) subdomains demarcated by the lipodystrophy protein, Seipin, lack of which impairs adipogenesis. However, the mechanisms of how these domains are selected is not completely known. These ER sites undergo ordered assembly of proteins and lipids to initiate LD biogenesis and facilitate establishment of ER-LD contact sites, a prerequisite for proper growth and maturation of droplets. LDs retain both physical and functional association with the ER throughout their lifecycle to facilitate bi-directional communication, such as exchange of proteins and lipids between the two organelles at these ER-LD contact sites. In recent years several molecular tethers have been identified that bridge ER and LDs together including few proteins that are found exclusively at these ER-LD contact interface. Here, we discuss recent advances in understanding the role of factors that ensure functionality of ER-LD contact site machinery for LD homeostasis.

## Introduction

Lipid droplets (LDs) are unique organelles found in all eukaryotic cells that serves as an energy reservoir. LDs store neutral lipids (NLs), predominantly triglycerides (TAG) and sterol esters (SE) inside its core enclosed by a phospholipid monolayer that recruits LD specific proteins, such as acyltransferases, lipases, and structural LD coat proteins (Olzmann and Carvalho, 2019). Mis-regulation of LD homeostasis results in several metabolic diseases, such as obesity, type-2 diabetes, insulin resistance, neuronal diseases, cancer, and lipodystrophy (reviewed in (Islimye et al., 2022; Kumari et al., 2023; Zadoorian et al., 2023). LDs are implicated in various cellular processes beyond lipid metabolism (Welte and Gould, 2017). Therefore, LD biogenesis, and its interaction with several other cellular organelles have been greatly explored in recent years and have provided novel insights. Endoplasmic reticulum (ER) localized NL synthesizing proteins generate NLs that assimilate within the ER bilayer membrane at low concentrations, however, upon reaching a certain threshold of few mol%, NLs coalesce into blisters/lenses as revealed by *in silico* and *in vitro* experiments (Hegaard et al., 2022; Khandelia et al., 2010; Thiam and Ikonen, 2021), and visualized as 30–60 nm structures between the two leaflets of the ER bilayer by electron microscopy (EM) in yeast (Choudhary et al., 2015). These lenses trap more NLs and eventually grow into nascent LDs that evaginate toward the cytoplasm delineated by phospholipid monolayer derived from the cytoplasmic leaflet of the ER membrane (Choudhary and Schneiter, 2021; Olzmann and Carvalho, 2019; Walther et al., 2017). LDs always remain associated with the ER and mature by acquiring various LD-resident proteins, and production of NLs locally at the LD surface (Jacquier et al., 2011; Salo et al., 2016). In this review, we discuss how early steps of LD biogenesis is crucial to determine functional ER-LD contact site formation, focusing on factors that play pivotal role in maintenance of these ER-LD contacts to ensure dynamic remodeling of LDs in response to metabolic demand.

### ER-LD contact sites

Contact sites are distinct domains which bring two organelles in close vicinity and facilitate inter-organellar communication, including organelle biogenesis, positioning, transport of ions, and lipids (Scorrano et al., 2019). Several molecular tethers have been identified that bridge two organellar membranes into proximity, such as tricalbins, extended synaptotagmins (E-Syts), and vesicle-associated membrane-protein associated proteins (VAPs) involved in tethering ER to plasma membrane (PM) (Liu et al., 2024). However, contacts between LDs and other organelles, including the ER, mitochondria, peroxisomes, Golgi, vacuole/lysosome are unusual as it represents bridging between a bilayer membrane and the LD monolayer (Hugenroth and Bohnert, 2020; Renne and Hariri, 2021). Although, bilayer-monolayer contacts between LDs and other organelles are highly dynamic and depend on metabolic cues, the ER-LD contacts are established while LDs are born *de novo* and remain throughout the life cycle of LDs. There are two types of ER-LD contact sites: (i) lipidic/membrane bridge at ER-LD continuum between ER bilayer and LD monolayer maintained via Seipin and (ii) contact site where ER juxtaposed to LDs is held together via protein tethers (Schuldiner and Bohnert, 2017). The interface at ER-LD contact sites requires factors not typically found in other organellar contacts. These proteins include LD biogenesis factors, and tether machineries including lipid transfer proteins (Hugenroth and Bohnert, 2020) (see Table 1 for a description of ER-LD contact site proteins). These lipidic bridges perhaps resemble remnant scars of LD budding and facilitate targeting of ER-resident proteins such as DGAT2, ACSL3 and GPAT4 onto LDs (Kassan et al., 2013; McFie et al., 2014; Wilfling et al., 2013). Once a functional ER-LD contact site is established, proteins can physically interact across the opposing membranes to support growth and expansion of LDs. Hence, a functional ER-LD contact regulates efficient induction of LD biogenesis, growth and maintenance of LDs, and its regression.

**TABLE 1:** Factors maintaining ER-LD contact sites.

Name	Protein function	Key References
Seipin (Sei1/Fld1 in yeast)	Defines ER subdomains for LD biogenesis, localizes at ER-LD contact sites, sequesters DAG/TAG in its toroidal rings, facilitates cargo exchange between ER and LDs	Klug et al. (2021); Prasanna et al. (2021); Salo et al. (2016); Sui et al. (2018); Yan et al. (2018); Zoni et al. (2021)
Ldb16 (yeast specific)	Seipin partner protein in yeast	Grippa et al. (2015); Klug et al. (2021); Wang et al. (2014)
LDAF1/Promethin (Ldo16/Ldo45 in yeast)	Key Seipin partner protein that associates in the presence of TAG, Found at ER-LD contact sites, Upon growth of LDs LDAF1 dissociates from Seipin and translocate onto LD periphery	Castro et al. (2019); Chung et al. (2019); Eisenberg-Bord et al. (2018); Teixeira et al. (2018)
FITM2 (Yft2, Scs3 in yeast	ER membrane protein that transiently enriches at ER-LD contact sites, regulates local DAG level at LD biogenesis sites, facilitates proper emergence of LDs from ER	Chen et al. (2021); Choudhary et al. (2018); Choudhary et al. (2016); Choudhary et al. (2015); Goh et al. (2015); Hayes et al. (2017); Kadereit et al. (2008)
MCTP2 (Pex30 in yeast)	ER membrane shaping proteins that localizes to LD biogenesis sites and facilitates biogenesis of LDs and peroxisomes, cooperates with Seipin in LD formation, localizes to ER-LD contact sites	Ferreira and Carvalho (2021); Joshi et al. (2016); Joshi et al. (2018); Joshi et al. (2021); Wang et al. (2018)
Snx14 (Mdm1 in yeast)	Promotes ER-LD tethering, aids ACSL3 during TAG synthesis and LD expansion	Datta et al. (2019); Hariri et al. (2019)
VPS13A-D	Inter-organellar bulk flow of lipids, localizes at ER-LD contact sites	Chen et al. (2022); Kumar et al. (2018)
ATG2	Acts as a lipid transfer protein regulating the size and number of LDs	Korfhage et al. (2023); Tamura et al. (2017)
DGAT2-FATP1	DAG acyltransferase, catalyzes formation of TAG, colocalizes with Seipin defined sites of LD biogenesis in the ER, relocalizes to mature LDs via ER-LD interface, FATP1 in the ER interacts with DGAT2 on LDs for enhanced growth of LD by siphoning acyl-CoA from ER to LDs	Choudhary et al. (2020); Jacquier et al. (2011); Malis et al. (2024); Xu et al. (2012)
Rab18-DFCP1	Promotes LD expansion by facilitating lipid transfer to nascent LDs and prevents lipolysis of mature LDs	Gao et al. (2019); Lukmantara et al. (2022); Martin et al. (2005); Ozeki et al. (2005)
MOSPD2	LD-ER tethering protein that facilitates lipid transfer activity and protein-membrane interaction	Di Mattia et al. (2018); Zouiouich et al. (2022)
Arf1/COPI	Remodels LD surface facilitating recruitment of TAG lipases and aids in lipolysis resulting in budding of tiny LDs	Wilfling et al. (2014)

### Lipid environment at ER-LD contact sites

The local lipid environment at LD biogenesis sites is not completely known. However, recent studies suggest that ER subdomains at which LDs form is likely to have locally enriched lipids, whose biophysical and biochemical properties facilitate the recruitment of LD biogenesis factors and assembly of LDs (Ben M’barek et al., 2017; Choudhary et al., 2020; Choudhary et al., 2018; Choudhary and Schneiter, 2020; Santinho et al., 2020). LD monolayer is derived from the ER membrane and is enriched in phospholipids (PLs), PC, PI, and PE, whereas PA and PS are relatively low (Leber et al., 1994; Tauchi-Sato et al., 2002). This suggests that PL composition of ER membrane and LD monolayer are not alike. Therefore, ER-LD contact interface perhaps plays a crucial role in divergence of PL composition of the two compartments, ER and LDs. The mechanistic details of this process is not well understood. The intrinsic molecular curvature of ER phospholipids have recently been suggested to modulate LD biogenesis and formation of ER-LD contacts (Chorlay and Thiam, 2018; Choudhary et al., 2018). An asymmetrical distribution of lipids at LD biogenesis sites is critical for regulating the directionality of LD emergence, as it alters surface tension, membrane curvature properties, and lateral pressure that govern how a droplet would bud from the ER (Chorlay et al., 2019; Chorlay and Thiam, 2018). Lipids that induce negative curvature such as diacylglycerol (DAG), and PE favour the embedded state of LDs in the ER membrane, however, lipids that promote positive membrane curvature such as lysophospholipids, stabilize an emerged state of LDs (Ben M’barek et al., 2017; Choudhary et al., 2018; Graff and Schneiter, 2024). *In vitro* studies with giant unilamellar vesicles (GUVs) containing an embedded artificial LD, indicates that it emerges towards the side having higher coverage of proteins and phospholipids, resulting in LDs with reduced surface tension (Chorlay et al., 2019). Interestingly, for a droplet to emerge fully toward the cytoplasm, a continuous supply of phospholipids within the cytoplasmic side of the ER is crucial to support the growing LD monolayer. Consistent with this notion, conditions when LDs expand dramatically upon oleate addition, the capacity to replenish phospholipids can become overwhelmed, that results in aberrant ER-LD contacts and as a result LDs bud towards the ER lumen (Chorlay et al., 2019). These aberrant LDs in the ER membrane will likely have an impaired recruitment of cytosolic factors onto LD surface, that manifests in ER stress and dysregulated LD homeostasis.

## Factors maintaining ER-LD contact sites

### Seipin

**FIGURE 1:**
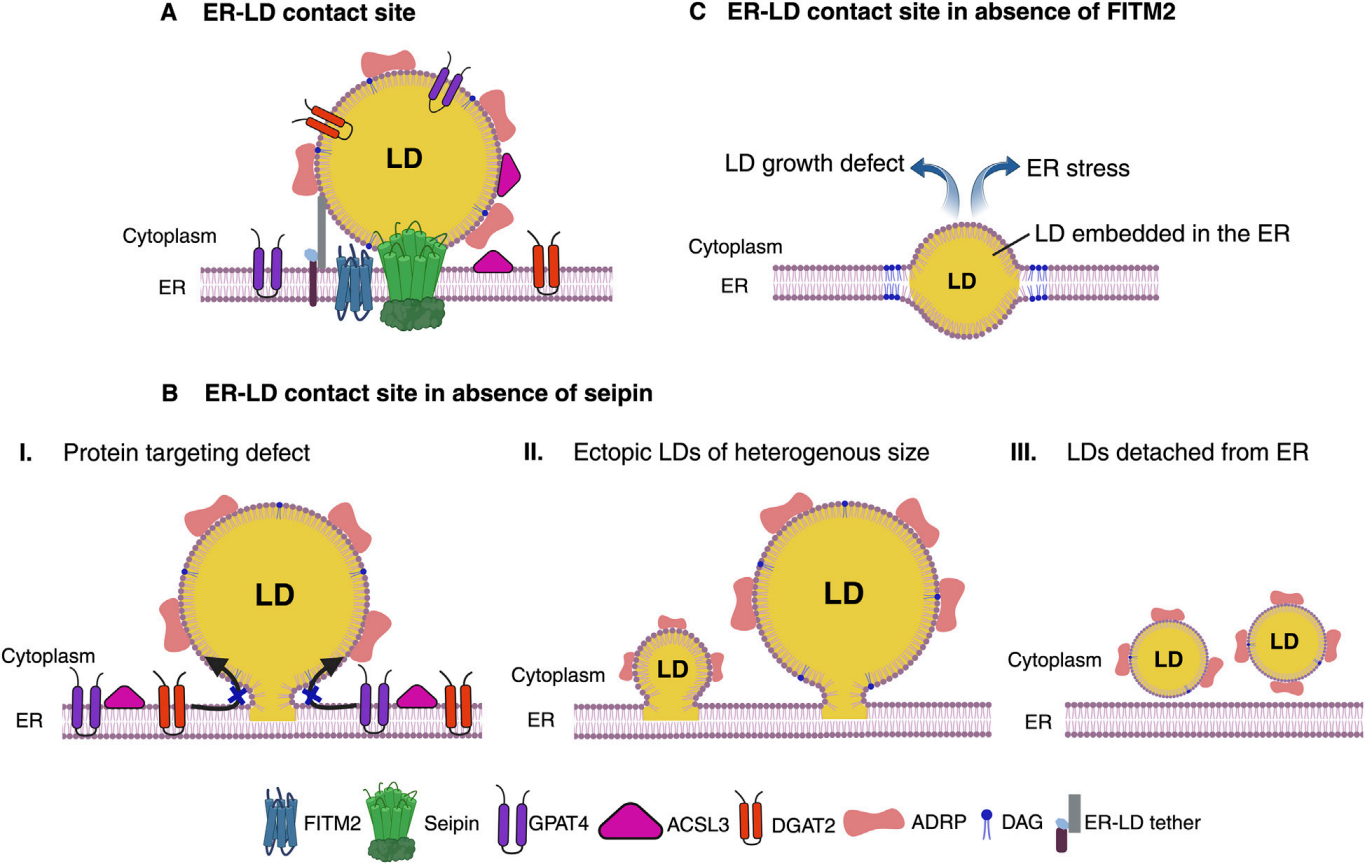
Functional ER-LD contact site. **(A)** Seipin defines ER subdomains to establish functional ER-LD contact site. ER membrane protein Seipin forms oligomeric ring complex that demarcates ER subdomains and acts as a scaffold for recruitment of LD biogenesis factors for droplet assembly. LDs grow and mature while remaining associated with Seipin at ER-LD contact site. A functional ER-LD contact site facilitates bi-directional partitioning of dually localized proteins, DGAT2, GPAT4, ACSL3 and lipids between the two compartments ER and LDs. FITM2, a polytopic ER membrane protein colocalizes with Seipin upon induction of *de novo* LD biogenesis. FITM2 proteins at these ER-LD contact sites regulates DAG levels. Molecular tethers can bridge ER and LDs for rapid flux of lipids between the two organelles. **(B)** Absence of Seipin impairs ER-LD cargo exchange. Lack of Seipin results in failure to prime LD biogenesis sites in the ER to recruit LD biogenesis factors and causes mis-localization of LD surface proteins (I). This results in random TAG production and formation of tiny, clustered, or supersized LDs at ectopic locations in the ER (II). LDs in Seipin deficient cells tend to detach from the ER and/or bud toward the ER lumen (III). **(C)** LD emergence defect in the absence of FITM2. Absence of FITM2 impairs droplet emergence from ER and therefore results in LDs being embedded in the ER membrane. An ER embedded droplet causes disrupted ER-LD contact site. These LDs fail to grow and manifests in ER stress. Figure created with BioRender.com with an academic license.

Amongst the known LD biogenesis factors, Seipin (Sei1/Fld1 in yeast) is probably the most extensively studied protein that plays a crucial role in defining ER subdomains of LD biogenesis, and is perhaps the first factor in the cascade of assembly of ER-LD contact site machinery (Choudhary and Schneiter, 2021; Salo, 2023; Salo et al., 2019) (Table 1). In mammals, the protein Seipin is encoded by the Berardinelli-Seip Congenital Lipodystrophy type 2 (BSCL2) gene, and its loss-of-function results in severe lipodystrophy in humans characterized by selective loss of adipose tissue, and seipinopathy, neurological disorders affecting motor neurons, which garnered an immense interest in its role in LD biogenesis (Cartwright and Goodman, 2012; Magre et al., 2001; Szymanski et al., 2007; Windpassinger et al., 2004). Seipin localizes to ER subdomains independently of NL synthesis or the presence of LDs, however, *de novo* production of LDs results in the recruitment of LD biogenesis factors, and enrichment of precursor lipids at these sites that drives assembly of nascent droplets, that grows and eventually buds toward the cytosol (Choudhary et al., 2020; Choudhary and Schneiter, 2020). Although Seipin is crucial for defining ER-LD contacts, the factors that determine its localization and regulation remain to be uncovered. Seipin remain associated with LDs at ER-LD contact sites in both yeast and mammalian cells (Fei et al., 2008; Salo et al., 2016; Wang et al., 2016). Consistent with this, removal of Seipin from these ER subdomains results in delayed LD formation and a diverse LD morphology defect, including a few supersized LDs and numerous tiny or clustered LDs, many of which are not functionally connected with the ER, resulting in an altered LD surface proteome (Cartwright et al., 2015; Grippa et al., 2015; Salo et al., 2016; Wang et al., 2016). Hence, a functional ER-LD contact is necessary for growth and maturation of LD by allowing transport of lipids between ER and LD at these contact sites, and to facilitate translocation of LD-resident proteins via ER-LD interface (Figure 1). Intriguingly, in worms homozygous deletion of Seipin manifests in embryonic lethality as a result of defective embryonic eggshell formation, implicating a key role of Seipin in embryonic development (Bai et al., 2020).

Recently resolved Cryo-EM structures of Seipin revealed novel insights about its functioning. Human, Fly and yeast proteins assemble into membrane embedded oligomeric ring complex having 11, 12, and 10 units respectively (Arlt et al., 2022; Klug et al., 2021; Sui et al., 2018; Yan et al., 2018). Interestingly, luminal domain of human and fly Seipin contains hydrophobic helix (HH) that orients toward inner leaflet of the ER membrane and displays affinity toward TAG binding. Molecular dynamic simulations (MDS) revealed key serine residues, S165, and S166 within the HH region of Seipin ring that interacts with DAG and TAG molecules thereby assisting in nanoscale NL clustering and aiding in lens formation (Prasanna et al., 2021; Zoni et al., 2021). However, this HH is missing in the yeast Seipin luminal domain, regardless, TAG binding activity is provided by the TMD region of Ldb16, a yeast specific Seipin partner protein that associates into a stable Seipin/Ldb16 complex (Klug et al., 2021). Lack of either Seipin or Ldb16 results in a similar LD morphology defect that can be rescued by overexpression of human Seipin (Grippa et al., 2015). Seipin preferentially localizes to tubular ER regions and physically stabilizes the ER-LD contact sites displaying a consistent membrane architecture with a diameter of ∼15 nm. Seipin can trap DAG and/or TAG at LD biogenesis sites thereby preventing its outward diffusion into the ER bilayer, indicating why LDs appear at Seipin-delineated areas and why Seipin knockout (KO) cells have higher TAG levels (Klug et al., 2021; Salo et al., 2019). Consistent with this, Seipin marked ER subdomains display enrichment of DAG as visualized by an ER-DAG sensor (Choudhary et al., 2020).

The outer layer of Seipin ring comprises of closely interacting beta-sandwich folds having similarity to lipid binding domains, and binds anionic phospholipids including PA *in vitro* (Yan et al., 2018). In agreement with this, Seipin has been implicated in regulating PA metabolism (Pagac et al., 2016; Sim et al., 2020). Moreover, Seipin prevents ectopic accumulation of PA in the ER, which triggers aberrant LD formation and interferes in the regulation of PPAR-gamma, thereby impairing adipogenesis (Rao and Goodman, 2021). In addition, PI(3)P, an anioninc phospholipid was recently shown to accumulate in foci in the ER in the absence of Seipin (Lukmantara et al., 2022). Interesting, reducing PI(3)P levels rescued the LD biogenesis defect of Seipin mutants (Lukmantara et al., 2022). Hence, Seipin acts as a key player in regulating local lipid composition at ER-LD contact sites.

### Seipin interacts with Promethin/LDAF1 at ER-LD contact sites

Promethin/Lipid Droplet Assembly Factor 1(LDAF1) has recently been identified in humans as a widely conserved protein forming a large ∼600 kDa hetero-oligomeric complex with Seipin (Castro et al., 2019; Chung et al., 2019). LDAF1 shows homology to yeast LD Organization (Ldo45, Ldo16) proteins (Eisenberg-Bord et al., 2018; Teixeira et al., 2018) (Table 1). Endogenous expression of LDAF1 results in punctate distribution in the ER, many of which colocalizes with Seipin, while free foci of both Seipin and LDAF1 also co-exist. However, upon induction of LD biogenesis, formation of *de novo* LDs occurs at sites that are positive for both Seipin and LDAF1, suggesting crucial role of both these proteins in defining sites of LD biogenesis in the ER (Chung et al., 2019). Consistent with this observation, redistribution of LDAF1 to ER-PM contact sites resulted in Seipin also being recruited at these sites and LDs being formed at these ectopic ER subdomains (Chung et al., 2019). Moreover, it has been shown that Seipin-LDAF1 complex co-purifies with TAG, however, Seipin alone does not, suggesting that the complex can trap TAG more efficiently than by Seipin alone (Chung et al., 2019). Given that Seipin-LDAF1 oligomeric complex might contain ∼66 transmembrane domains together, such an arrangement of hydrophobic helices may serve to efficiently sequester TAG, thereby allowing its nucleation into droplets (Chung et al., 2019). Remarkably, loss of Seipin results in mislocalization of LDAF1, suggesting that previous studies related to lack of Seipin, might in-fact represent conditions where both Seipin and LDAF1 were absent (Chung et al., 2019). Upon LD expansion, LDAF1 dissociates with Seipin and translocates onto LD periphery, while Seipin remains localized at ER-LD contact site, suggesting a hairpin type of topology of LDAF1 that allows it to reside in the ER bilayer and also associate with the LD monolayer surface at ER-LD contact site (Chung et al., 2019). Recently it has been shown that yeast Seipin and its partner Ldb16 are also involved in packaging of LDs containing exclusively of SEs (Renne et al., 2022). Moreover, presence of TAG facilitates nucleation of SE containing LDs at Seipin enriched ER subdomains (Dumesnil et al., 2023). Hence, Seipin oligomeric complex indeed creates a platform in the ER for functional ER-LD contact formation.

### Seipin creates a diffusion barrier at ER bilayer-LD monolayer interface

Presence of Seipin at ER-LD contact site appears to be crucial for regulating the flow of NLs between ER and LDs, thereby coordinating continuous LD growth and preventing ripening, a phenomenon where larger LDs would grow at the expense of shrinking smaller LDs (Salo et al., 2019). Hence Seipin counteracts ripening and allows uniform growth of LDs during LD biogenesis, a process that gets perturbed in the absence of Seipin. Interestingly, Seipin also facilitates the movement of proteins between ER and LDs at ER-LD contact sites perhaps by physically stabilizing these interfaces (Grippa et al., 2015; Salo et al., 2016). Lack of Seipin results in mislocalization of LDAF1 in cells (Chung et al., 2019), Pex30, an ER shaping protein (Joshi et al., 2018), and lipolytic enzymes Tgl1, and Yeh1 in yeast (Grippa et al., 2015). Recently it has been shown that ER-LD contact site constitutes a diffusion barrier for integral ER membrane proteins and excludes these proteins to diffuse onto LD monolayer, such as Sec61, a subunit of ER translocon complex, and Wbp1, a subunit of oligosaccharyltrasferase glycoprotein complex (Khaddaj et al., 2022). However, proteins having hairpin topology such as mammalian DGAT2, and its yeast homolog Dga1 that catalyzes last step of TAG synthesis, partition from ER to LD periphery via membrane bridge at ER-LD continuum (Jacquier et al., 2011; Stone et al., 2009). Similarly, glycerol-3-phosphate acyltransferase 4 (GPAT4), which catalyzes the rate-limiting step of TAG biosynthesis relocalizes from ER to LD monolayer at ER-LD interface to coordinate LD growth (Wilfling et al., 2013). Interestingly GPAT4 positive LDs showed enrichment of acyl-CoA synthetase enzymes, ACSL1 and ACSL3, which activates fatty acids, a substrate for TAG-synthase enzymes, as well as AGPAT3, an enzyme that would consume the product of GPAT4 reaction (Wilfling et al., 2013). A stable ER-LD bridge is crucial for the transport of these ER-resident proteins onto LDs. Consistent with this, recently it has been shown that LD-resident proteins equilibrate in a continuous and bi-directional fashion between two mating yeast cells upon zygote formation via the interconnected ER network (Cottier and Schneiter, 2022). Remarkably, this protein transfer is affected in the absence of Seipin, indicating that Seipin regulates cargo exchange at bilayer-monolayer interface (Cottier and Schneiter, 2022). Recently, *in vitro* studies using microfluidics to reconstitute LDs with or without proteins inside a membrane bilayer resulted in asymmetric lens-like LD structure when proteins were included (Puza et al., 2022). Using fluorescence recovery after photobleaching (FRAP) on LD surface resulted in a reduced exchange of PE and PC phospholipids between LD monolayer and membrane bilayer interface, indicating the presence of a diffusion barrier, that likely affects the distribution of membrane proteins (Puza et al., 2022).

### Fat storage-inducing transmembrane proteins (FITM)

A functional ER-LD contact site recruits several LD biogenesis factors. One such factor is FITM. FITM proteins are widely conserved ER transmembrane proteins implicated in LD formation (Kadereit et al., 2008). Mammals express two FITM proteins, FITM1 and FITM2 having 50% similarity to each other. FITM1 is primarily expressed in heart and skeletal muscles while FITM2 shows ubiquitous expression with elevated expression in white and brown adipose tissue (Kadereit et al., 2008). Strikingly, overexpression of FITM2 in 3T3-L1 fibroblasts results in accumulation of TAG loaded LDs, and its absence leads to decrease in LD number and size, suggesting a crucial role of FITM2 in LD biogenesis (Kadereit et al., 2008). Intriguingly, overexpression of FITM2 does not affect the rate of TAG synthesis, nor does it alter the expression of genes associated with TAG production, or perturb NL turnover, hence implicating that FITM2 directly acts in the partitioning of TAG into storage LDs affecting TAG distribution in the ER (Kadereit et al., 2008). In line with this hypothesis, it has been shown that purified FITM2 binds to TAG and its precursor DAG *in vitro* (Gross et al., 2011). Impairment of FITM2 function is linked with metabolic disorders such as type-2 diabetes, insulin resistance, lipodystrophy, and non-metabolic diseases such as deafness-dystonia syndrome (Miranda et al., 2014; Zazo Seco et al., 2017; Zheng et al., 2022). Both FITM1 and FITM2 possess six transmembrane domains (TMD) with N- and C-terminal exposed toward the cytoplasm and conserved histidine residues (H155, and H214) within the TM-4 and TM-5 segments oriented towards the ER lumen (Hayes et al., 2017). FITM2 is more widely conserved than FITM1, since yeast, fly, and worm have only orthologues of FITM2. Budding yeast encodes for two FITM2 homologs (Kadereit et al., 2008), Scs3, a protein previously reported to be involved in inositol prototrophy (Hosaka et al., 1994) and Yft2. Lack of Scs3 results in inositol auxotrophy and defects in ER membrane homeostasis (Moir et al., 2012).

A striking defect of FITM2 depletion, the embedding of LDs in the ER membrane has been observed from yeast to humans, suggestive of abortive step in the process of LD budding from the ER and that FITM2 perhaps aids in this process (Choudhary et al., 2015; Goh et al., 2015) (Figure 1). Remarkably, deletion of FITM2 in worm and mice is lethal, implying that FITM2 fulfils an important function in lipid metabolism (Choudhary et al., 2016; Choudhary et al., 2015; Goh et al., 2015). It has been suggested that FITM2 proteins could perhaps harbor lipid phosphatase/phosphotransferase activity on the ER lumen side and mutating conserved histidine residues perturbs FITM2 activity *in vivo* (Hayes et al., 2017). Moreover, recently it has been shown that FITM2 can catalyze cleavage of acyl-CoA to acyl 4′-phosphopantetheine *in vitro*, and that this activity might be important for maintaining proper ER membrane structure, as absence of FITM2 leads to ER whorls and affects lipid homeostasis (Becuwe et al., 2020).

Despite these advances the mechanisms by which FITM2 regulates LD emergence or pathways that get perturbed resulting in lethality remain yet to be determined. The fact that yeast FITM2, Yft2 becomes enriched at ER-LD contact sites upon induction of *de novo* LD formation (Choudhary et al., 2020; Choudhary et al., 2018), implicates its role in condensation/sequestration of TAG and/or DAG at sites of LD biogenesis in the ER marked by Seipin protein (Table 1). Consistent with this, Yft2 puncta at LD biogenesis sites co-enriches with ER DAG-sensor, a probe to visualize DAG distribution in the ER (Choudhary et al., 2020; Choudhary et al., 2018). Therefore, FITM2 proteins might be involved in modulating DAG and/or TAG levels locally at LD biogenesis sites. One possibility could be that since they have affinity for DAG/TAG, they might act as sinks of DAG/TAG at these sites, hence preventing overaccumulation of these NL that could be detrimental for proper LD budding and establishment of functional ER-LD contact sites (Figure 1). In the absence of FITM2, perhaps DAG levels at LD biogenesis sites might be too high, and owing to negative intrinsic curvature of this class of phospholipid it could perturb proper LD budding promoting embedded state of droplets in the ER (Choudhary et al., 2018). In line with this, increasing positive curvature phospholipids, such as lysophosphatidylcholine (LysoPC) alleviates this LD emergence defect (Choudhary et al., 2018).

Moreover, FITM2 interacts with ER tubule forming proteins in mammalian cells, such as Rtn4, REEP5 and cytoskeleton protein Septins, thereby stabilizing the oil lens curvature and facilitating LD budding (Chen et al., 2021). Lack of these ER tubulating proteins results in fewer and smaller LDs, suggesting that ER membrane curvature plays an important role in LD biogenesis (Chen et al., 2021). Another study implicates the role of FITM2 in lipidation of very low-density lipoprotein (VLDL) particles. Lack of FITM2 in both *in vitro* and *in vivo* hepatic cells leads to secretion of TAG depleted VLDL resulting in ER stress (Wang et al., 2023). In the coming years, further work related to FITM proteins will provide novel insights into how they function in maintaining ER-LD contacts and elucidating their role in lipid metabolism.

### ER membrane shaping protein Pex30/MCTPs

Growing evidence suggests that ER shape plays a crucial role in LD biogenesis. Tubular ER region is more permissive for LD formation than ER sheets, owing to high curvature stress (Santinho et al., 2020). Therefore, factors that mediate tubulation of ER, such as reticulons, atlastins, and REEPs have been implicated in LD biogenesis (Klemm et al., 2013; Renvoise et al., 2016). Recently, a novel family of ER tubulating proteins Pex30 in yeast and its human homolog Multiple C2 and transmembrane domain containing 2 (MCTP2), has been shown to play an important role in LD biogenesis (Joshi et al., 2018; Wang et al., 2018). These proteins harbor N-terminal reticulon homology domain (RHD), a hairpin-type of membrane shaping domain found in reticulons. Importantly, it has been demonstrated that Pex30/MCTP2 tubulate subdomains of the ER at which biogenesis of both LDs and peroxisomes occur (Joshi et al., 2018; Wang et al., 2018). These Pex30 containing ER sites are distinct from those of ER exit sites at which COP-II coated vesicles originate (Joshi et al., 2016). Remarkably, Pex30 remains associated with mature LDs at ER-LD contact sites like Seipin. Absence of sole MCTP2 protein in worms leads to reduced LD number and size compared to WT animals, however, it does not result in lethality (Joshi et al., 2018). Recently MCTP1, another member of MCTP family was shown to deform ER subdomains and facilitate LD biogenesis, like MCTP2 (Joshi et al., 2021). Both these proteins mark LD biogenesis sites, colocalize with Seipin, have lipid binding affinity, and are most likely involved in organizing ER-LD contact site formation (Joshi et al., 2021).

The membrane deformation activity of Pex30/MCTP proteins at LD biogenesis sites might be important to accommodate DAG and/or TAG (Table 1). In agreement with this, Pex30 colocalizes with DAG-enriched ER sites containing LD biogenesis factors, Seipin and Nem1, a regulator of DAG production (Joshi et al., 2018). In Seipin deficient cells, Pex30 is mislocalized to a single punctum, however, deletion of Pex30 does not impair Seipin localization, but these Seipin foci fail to recruit TAG synthesizing enzymes and hence are nonfunctional (Choudhary et al., 2020; Joshi et al., 2018; Wang et al., 2018). Moreover, a double deletion of both Pex30 and Seipin results in an additive growth defect, dysregulated LD formation, and elevated DAG levels that perhaps disfavors emergence of LDs, resulting in global TAG accumulation in the ER membrane (Joshi et al., 2018; Wang et al., 2018). Thus, Pex30 plays a crucial role at LD assembly sites by tubulating these regions, and hence driving the recruitment of various factors at these domains for nucleation of LDs.

### Sortin nexin 14 (Snx14)

Another important protein at ER-LD contact site is sortin nexin 14 (Snx14) in humans, and its orthologue Mdm1 in yeast, providing novel insights into how these contacts function in regulating LD biogenesis (Datta et al., 2019; Hariri et al., 2019). Loss-of-function mutations in the ER-resident protein Snx14 is the cause of spinocerebellar ataxia autosomal recessive 20 (SCAR20) characterized by intellectual impairment, cerebellar atrophy, ataxia and defective speech development (Thomas et al., 2014). Snx14 is an ER membrane protein that associates with LD surface in trans via its amphipathic helix present in the C-Nexin domain found in its C-terminus (Datta et al., 2019). Consistent with functions in regulating ER-LD contact sites, overexpression of Snx14 dramatically expands ER-LD tethering, whereas lack of Snx14 reduces these interface (Datta et al., 2019). Snx14 shows a uniform distribution in the ER in absence of exogenous fatty acids (FA). However, upon FA supplementation, Snx14 enriches at ER-LD contact sites and colocalizes with fatty acyl-CoA synthetase ACSL3, suggesting increased demand of activated FA to be incorporated into TAG at ER-LD contact sites, and implying that these two proteins might cooperate in LD expansion (Table 1). In agreement with this, absence of Snx14 sensitizes cells to FA induced lipotoxicity (Datta et al., 2019). Recent studies in mice have shown that Snx14 deficiency causes embryonic lethality, delayed development, compromised lipid storage, defective metabolism and lipotoxicity, suggesting a crucial role of ER-LD contacts in dynamic remodeling of LD contents (Zhou et al., 2024).

### Vacuolar protein sorting family member 13 (VPS13) and autophagy-related protein 2 (ATG2)

VPS13 belongs to a highly conserved family of lipid transfer proteins, having four members in humans, VPS13A-D, whereas a single member in yeast, Vps13 (Kumar et al., 2018) (Table 1). These proteins adopt a rod like extended bridge conformation, consisting of a hydrophobic groove that enables bulk flow of lipids, such as phospholipids at multiple membrane contact sites, including ER-LD contacts (Chen et al., 2022; Kumar et al., 2018). The yeast Vps13 is more closely related to mammalian VPS13A and VPS13C. Mutations resulting in loss-of-function of VPS13A and VPS13C manifests in neurological diseases, such as chorea acanthocytosis and Parkinson’s disease respectively (Lesage et al., 2016; Ueno et al., 2001). VPS13A is also found at ER-mitochondria contacts, whereas VPS13C tethers ER-late endosomes/lysosome contacts (Kumar et al., 2018). Both VPS13A and VPS13C are found at ER-LD contact sites and VPS13C is detected in LD proteomes (Bersuker et al., 2018). VPS13 proteins have a conserved domain organization, comprising of chorein domain found in N-termini, a putative WD40 module, a DH-like domain, and a PH domain. VPS13 N-termini domain contains a small VAP-interacting motif, FFAT (two phenylalanines in an acidic tract) that interacts with VAMP-associated protein (VAP), a key ER transmembrane protein that works as an anchor for many contact site machineries (Kumar et al., 2018). Absence of VPS13A FFAT motif or lack of VAP results in localization of VPS13A to mitochondrial surface, without any ER targeting. Upon overexpression, both VPS13A and VPS13C (Kumar et al., 2018) as well as VPS13D (Wang et al., 2021) interacts with LDs via their amphipathic helices found in C-terminal end of the protein, involving DH-like/PH domain region and localize at ER (VPS13A, VPS13C) or mitochondria (VPS13D) contacts. Recently deciphered cryo-EM structure of N-terminal fragment of VPS13 revealed it can form a channel lined with hydrophobic residues that allows bulk flow of lipids between two opposing membranes at contact sites (Li et al., 2020).

ATG2 is an essential autophagy protein containing conserved chorein domain in its N-termini, localizes at ER-LD contact sites, and thereby regulates the size and number of LDs by acting as a lipid transfer protein at these contact sites (McEwan and Ryan, 2022; Tamura et al., 2017) (Table 1). Apart from ER-LD contact sites, ATG2 localizes to autophagosomes and plays a crucial role in lipid transfer to enlarging autophagosomes at ER-autophagosome contact sites (Velikkakath et al., 2012). Lack of ATG2 results in blocked autophagic flux represented by unclosed autophagic structures, and aggregated large LDs in an autophagy independent manner (Velikkakath et al., 2012). A recent study has shown that ATG2 gets preferentially recruited to LD monolayer, and purified ATG2 can drive transfer of phospholipids in artificial LDs within a bridge-like conduit (Korfhage et al., 2023). Mutant protein which blocks lipid transport *in vitro* can still localize to LDs but cannot prevent the aggregation of LDs in ATG2 KO cells (Korfhage et al., 2023). Taken together, VPS13 members including ATG2 apart from tethering also act as large conduits facilitating bulk lipid transport between two organellar membranes.

### FATP1-DGAT2

In mammals, diacylglycerol O-acyltransferase 2 (DGAT2) encodes one of the two enzymes that catalyzes the last step of TAG production in which DAG is covalently bound to fatty acyl-CoA (Cases et al., 2001). DGAT2 contains a single hydrophobic hairpin motif that anchors it to the ER bilayer, however, upon LD expansion the protein can translocate onto the LD monolayer (Jacquier et al., 2011; Stone et al., 2006). Fatty acyl-CoA synthetase, FATP1, primarily found in the ER interacts directly with DGAT2 on LD surface upon droplet expansion and both these proteins show enrichment at the ER-LD contact sites (Xu et al., 2012). Immunoelectron microscopy revealed enrichment of both proteins at ER-LD contact sites. FATP1-DGAT2 collectively form a complex connecting ER and LDs and their overexpression leads to enhanced LD growth by siphoning acyl-CoA from ER to LDs for TAG synthesis (Xu et al., 2012) (Table 1). Consistent with this, loss of FATP1 or DGAT2 inhibited LD expansion in *C. elegans* (Xu et al., 2012). A recent study has shown that Rab1b, a member of the small GTPase family of proteins and a key protein in ER-to-Golgi transport is crucial for LD maturation. The active form Rab1b-GTP is essential for LD development and targeting DGAT2 to LD surface at later stages of LD biogenesis at ER-LD contacts (Malis et al., 2024).

### Rab18 and DFCP1

Several Rab GTPase family proteins have been implicated in LD dynamics (Li and Yu, 2016). Rab8a in association with Fsp27 is involved in LD fusion and growth, whereas Rab7 interacts with Rab-interacting lysosomal protein (RILP) and promotes lipophagy (Schroeder et al., 2015; Wu et al., 2014). Rab18 has been shown to localize to both ER and LDs, however, upon overexpression, both light and immuno electron microscopy revealed tight apposition between ER and LDs (Martin et al., 2005; Ozeki et al., 2005). Interestingly, Rab18 localized on a subset of LDs that were devoid of PLIN2/ADRP. Furthermore, upon oleate addition, newly forming droplets were lacking Rab18, however, after 3 h of induction Rab18 started to decorate a subset of LDs (Martin et al., 2005). This suggests that Rab18 gets recruited to mature LDs rather than nascent LDs undergoing biogenesis. In support of this, Rab18 showed increased localization with LDs when cells were forced to undergo lipolysis, whereas blocking lipolysis abrogated this association (Martin et al., 2005). In line with this, larger LDs accumulate in fibroblasts taken from patients suffering from mutation in Rab18 called Warburg Micro syndrome further suggests evidence for a role of Rab18 in LD homeostasis (Carpanini et al., 2014). In the absence of Rab18, LD biogenesis does not seem to be impaired, however, small LDs of ∼100 nm size start to accumulate that fail to grow, suggesting important role of Rab18 at ER-LD contact site that regulates LD dynamics (Xu et al., 2018). Interaction of Rab18 with the ER is mediated by the NRZ tethering complex factors that associate with the ER membrane SNARE protein Use1 (Xu et al., 2018) (Table 1). Use1 is part of the ER SNARE complex together with BNiP1, SNARE syntaxin18, and Sec22b (Xu et al., 2018). COPI, TRAPII and Rab18 form complexes and regulates recruitment of Rab18 to LDs thereby regulating LD formation (Li et al., 2017).

An important interactor/effector of Rab18 is ER localized double FYVE-domain containing protein 1 (DFCP1). It has been shown that upon induction of LD formation, DFCP1 redistributes into puncta in the ER possibly at sites of TAG synthesis and representing nascent LDs (Gao et al., 2019). These puncta move along the ER and fuse to form expanding LDs which is controlled by Seipin (Li et al., 2019). This recruitment is independent of FYVE domain, however it requires the presence of Rab18. Interestingly, overexpression of DFCP1 resulted in increased LD size at the expense of total LD number, whereas knockdown has the opposite effect, suggesting role of DFCP1 in regulating LD growth via interaction with Rab18 (Li et al., 2019). Reduced association of DFCP with LDs was found in cells with Seipin deficiency. Deficiency of DFCP1 results in lipidation defect of initial LDs suggesting that DFCP1 facilitates lipid delivery to nascent LDs (Lukmantara et al., 2022). DFCP1 was found to co-immunoprecipitate with Rab18-GTP but not with Rab18-GDP (Gao et al., 2019) and mediates ER-LD contact formation (Li et al., 2019) (Table 1). Taken together, DFCP1-Rab18 complex at ER-LD contact sites plays a crucial role in LD expansion.

### MOSPD2

Recently a novel ER tether protein named motile sperm domain containing protein 2 (MOSPD2) has been identified. MOSPD2 is anchored in the ER via its C-termini transmembrane domain (Di Mattia et al., 2018). Lack of MOSPD2 impairs droplet assembly. MOSPD2 contains a Major Sperm Protein (MSP) domain in the cytoplasm which binds FFAT motif containing tethering proteins from mitochondria, golgi, and endosomes and mediates the formation of contact sites, and a CRAL-TRIO domain that possesses a putative lipid transfer activity (Di Mattia et al., 2018). Apart from its role as inter-organellar tethers, MOSPD2 has been shown by correlative light and electron microscopy (CLEM) to enrich at ER-LD contact sites, where it facilitates protein-membrane interaction (Zouiouich et al., 2022) (Table 1). Intriguingly, localization of MOSPD2 at ER-LD contact sites is independent of presence of Seipin or the presence of MSP domain (Zouiouich et al., 2022). Interestingly, CRAL-TRIO and transmembrane domain are necessary for MOSPD2 to get recruited at ER-LD interface, where an amphipathic helix in the CRAL-TRIO domain mediates interaction with the LD surface (Zouiouich et al., 2022). Taken together, MOSPD2 represent a novel ER tether that bridges ER-LD together to regulate LD homeostasis.

### Arf1/COPI

ADP-ribosylation factor 1 (Arf1) belongs to the conserved ARF superfamily of small GTPases. Arfs are soluble proteins, however, they associate with membranes due to N-termini myristoylation and function as regulators of vesicular trafficking and actin remodeling (Donaldson and Jackson, 2011). The coat protein complex I (COPI) consists of Arf1 and the heptameric coatomer protein complex. The Arf1/COPI coated vesicles regulates the retrograde vesicular trafficking from the Golgi to the ER (Spang, 2002). Interestingly, proteomic and cell culture studies have revealed Arf1/COPI machinery to localize on LDs (Bouvet et al., 2013; Nakamura et al., 2005). Surprisingly, unlike vesicular budding from bilayer membranes, it has been shown that GTP-bound Arf1/COPI proteins can interact with LD monolayer and facilitate budding of ∼60 nm TAG rich LDs from artificial droplets *in vitro* (Thiam et al., 2013). Budding of nano droplets *in vivo* depletes LD monolayer surface of phospholipids, thereby exposing TAG inside LD core, increasing surface tension that promotes association of various proteins with LDs (Thiam et al., 2013). Consistent with this, Arf1/COPI machinery mediates targeting of ATGL, a TAG lipase and ADRP, an LD resident protein onto LDs and their deficiency results in impaired lipolysis (Soni et al., 2009). Moreover, Arf1/COPI machinery establishes ER-LD membrane bridges by directly removing phospholipids which allows for the rapid localization of membrane-associated proteins such as ATGL, DGAT2, AGPAT3 and GPAT4 onto LD surface (Wilfling et al., 2014) (Table 1). Lack of Arf1/COPI machinery results in small-sized LDs due to impaired targeting of TAG synthesizing enzymes to growing LDs (Wilfling et al., 2014). Moreover, recently in yeast it has been shown that Arf1 localizes at bi- and tripartite organellar contacts involving LDs, mitochondria, and peroxisomes and regulates the transport of acetyl-CoA and FA from LDs to mitochondria in yeast. Expression of a hyperactive mutant Arf1-11 resulted in increased contact formation between ER, LDs and mitochondria where Arf1 plays a crucial regulator of all these contact sites (Enkler et al., 2023). Arf1 coprecipitates with TAG-synthase Dga1, a crucial regulator of ER-LD contact sites in yeast and mammals, and yeast TAG lipase Tgl4 and the acyl-CoA synthetase Faa1 (Xu et al., 2012). Arf1-11 overexpression resulted in aggregation of FA in LDs due to decreased expression of mitochondrial fatty acid transporters, leading to fragmented mitochondria that curtailed ATP synthesis (Enkler et al., 2023). Taken together, Arf1 as a tether regulates LD and mitochondrial dynamics.

## Concluding remarks

There has been an increased interest in understanding the molecular mechanisms of LD biogenesis and the role of factors that establish functional ER-LD contact sites. The architecture of ER-LD contact sites is strikingly different than classical membrane contact site between membrane bilayers of two organelles. These ER-LD contacts represent hemi-fusion between ER bilayer and LD monolayer membrane and act like bridges between these two organelles. Since LDs are formed *de novo* in contrast to other organelles, these ER-LD contacts are established during birth of LDs and ensure functional coupling between the two compartments throughout its life cycle. Several molecular tethers at ER-LD contacts are exclusive to these regions, while other classical contact site proteins also associate with ER-LD contacts. A major function of these ER-LD contact interface is to provide molecular highway for trafficking of lipids and proteins between the two compartments. In recent years our understanding of several tether components at ER-LD contacts have provided new insights about its functioning.

However, several outstanding questions awaits further investigation. How ER-LD contact machinery is regulated? Since Seipin defines sites of LD biogenesis in the ER, what determines the localization of Seipin? Finding answer to these questions will be crucial to decipher how ER-LD contacts are established at the very first place. During LD biogenesis some of the molecular tethers are selectively targeted to these ER-LD contact sites, whereas other tethers get recruited only when LDs are undergoing lipolysis. Hence, how does the metabolic state of the cell guide recruitment of these factors at ER-LD contact sites in a dynamic fashion? Our understanding of how dysregulated ER-LD contacts result in diseases is still far from complete. It would be interesting to explore how ER-LD contacts are exploited by pathogens for their benefit. ER-LD bridges act as barrier for ER transmembrane proteins to be trafficked to LD periphery, while allowing certain proteins with special hairpin topology to translocate onto LD surface. It would be interesting to determine if this holds true for all hairpin containing proteins or is there an exception to this rule? Another interesting area of research is to know which factors are responsible for severing ER-LD contacts to completely detach LDs when they undergo lipophagy. Once LDs are detached and free, these LDs cannot grow due to lack of vesicular trafficking between ER and LDs, and hence are destined for degradation, perhaps by lipophagy. Therefore, maintenance of ER-LD contacts is central for ER/lipid homeostasis. Hence better understanding of these processes will provide novel insights into the cell biology of LDs and pathophysiology of LD related disorders.

## Glossary

ACSL3: Acyl-CoA synthetase long-chain family member 3
ADRP: Adipose differentiation-related protein
AGPAT3: 1-acylglycerol-3-phosphate O-acyltransferase 3
Arf1: ADP-ribosylation factor 1
ATG2: Autophagy-related protein 2
ATGL: Adipose triglyceride lipase
BNIP1: BCL2 interacting protein 1
BSCL2: Berardinelli-Seip Congenital Lipodystrophy type 2
CLEM: Correlative light and electron microscopy
COPI: Coat protein complex I
DAG: Diacylglycerol
DFCP1: Double FYVE-domain containing protein 1
Dga1: Diacylglycerol acyltransferase
DGAT2: Diacylglycerol O-acyltransferase 2
EM: Electron microscopy
ER: Endoplasmic reticulum
FA: Fatty acids
Faa1: long-chain-fatty-acid-CoA ligase 1
FATP1: Fatty acyl-CoA synthetase
FFAT: two phenylalanines in an acidic tract
FITM: Fat storage-inducing transmembrane proteins
FRAP: Fluorescence recovery after photobleaching
Fsp27: Fat-specific protein 27
GDP: Guanosine diphosphate
GPAT4: Glycerol-3-phosphate acyltransferase 4
GTP: Guanosine triphosphate
GUV: Giant unilamellar vesicles
HH: Hydrophobic helix
kDa: kilo Dalton
KO: Knockout
LD: Lipid droplet
LDAF1: Lipid Droplet Assembly Factor 1
Ldb16: Low dye binding
Ldo: LD Organization
LysoPC: Lysophosphatidylcholine
MCTP: Multiple C2 and transmembrane domain containing 2
Mdm1: Mitochondrial Distribution and Morphology
MDS: Molecular dynamic simulations
MOSPD2: Motile sperm domain containing protein 2
MSP: Major Sperm Protein
NL: Neutral lipid
nm: nanometre
PA: Phosphatidic acid
PC: Phosphatidylcholine
PE: Phosphatidylethanolamine
Pex30: Peroxisome related protein
PL: Phospholipid PLIN2: Perilipin 2
PM: Plasma membrane
PS: Phosphatidylserine
Rab: Ras-associated binding proteins
REEP5: Receptor Expression Enhancing Protein 5
RHD: Reticulon homology domain
RILP: Rab-interacting lysosomal protein
Rtn4: Reticulon 4
SCAR20: Spinocerebellar ataxia autosomal recessive 20
Scs3: Suppressor of Choline Sensitivity
SE: Sterol esters
Sec: Secretory
SNARE: Soluble N-ethylmaleimide-sensitive factor activating protein receptor
Snx14: Sortin Nexin 14
TAG: Triglycerides
TMD: Transmembrane domain
VAMP: Vesicle-associated membrane protein
VAPs: Vesicle-associated membrane-protein associated proteins
VLDL: Very low-density lipoprotein
VPS13: Vacuolar protein sorting family member 13
Wbp1: WW domain binding protein 1
